# Modified coronally advanced flap for multiple anterior gingival recessions: Case report

**DOI:** 10.1002/cap.70049

**Published:** 2026-06-17

**Authors:** Kevimy Agossa, Dino Calzavara, Javier Calatrava, Hom‐Lay Wang

**Affiliations:** ^1^ Department of Periodontics and Oral Medicine University of Michigan School of Dentistry Ann Arbor Michigan USA; ^2^ Department of Periodontology School of Dentistry, University and CHU of Lille Lille France; ^3^ Section of Graduate Periodontology, Faculty of Odontology University Complutense Madrid Spain

**Keywords:** case report, connective tissue/transplantation, gingival recessions, gingivoplasty/methods, surgical flaps

## Abstract

**Background:**

The frontal envelope coronally advanced flap (CAF), designed for multiple adjacent gingival recessions (MAGR) of the anterior maxilla, involves tunneling of the midline papilla. However, when treating frontal and lateral MAGR in a single session, the tunneled midline papilla can act as a fixed point, which may limit coronal advancement or lead to mucosal folds in the gingival margin.

**Methods:**

This case report introduces a modification of the CAF technique in which a V‐shaped split‐thickness surgical papilla is elevated at the midline to enhance flap mobility. Two patients with combined maxillary frontal and bilateral types 1 and 2 (RT1, RT2) MAGR ranging from 2–6 mm were treated using this approach.

**Results:**

Postoperative healing was uneventful for both cases. In Case 1, complete and stable root coverage with excellent tissue integration and color match was observed at 18 months. In Case 2 (RT2 MAGR), near‐complete root coverage was achieved at 6 months.

**Conclusion:**

Within the limitations of this case report, the modified CAF appears to be an effective approach for managing MAGR extending beyond the incisors and involving both maxillary quadrants. Clinicians may consider this modification when enhanced coronal flap mobility is required to treat extensive frontal and lateral MAGR in a single session.

**Key points:**

- In the traditional frontal envelope coronally advanced flap, the tunneled midline papilla may act as a fixed point, which may restrict coronal advancement or lead to mucosal fold formation in the gingival margin.
- This case presents a modified design that enhances coronal flap mobility and achieves optimal root coverage in cases of extensive frontal and lateral maxillary anterior gingival recessions (MAGR).
- The success of this approach relies on midline papilla anatomy and precise incision placement, both of which support adequate flap stability, enhancing the predictability of root coverage outcomes.

**Plain language summary:**

Gum recession occurs when the gum margin moves downward, exposing part of the tooth root. When several neighboring teeth in the upper front region are affected, treatment can be particularly challenging because of the esthetic demands in this visible area. A commonly used surgical method, known as the coronally advanced flap (CAF), repositions the gum tissue to cover the exposed roots. However, in its traditional form, the tissue between the two front teeth is not lifted but tunneled, which can limit tissue movement and occasionally cause small folds along the gum line. This report describes a refined version of the CAF technique in which a small, V‐shaped flap is gently lifted between the front teeth to improve tissue mobility and adaptation. Two patients were treated using this approach, both achieving favorable healing, stable root coverage, and a natural appearance that blended well with surrounding tissues. This modification may help clinicians manage wider areas of gum recession in the upper front region while maintaining esthetic harmony.

## INTRODUCTION

Gingival recession (GR) refers to the apical migration of the gingival margin, resulting in root surface exposure beyond the cementoenamel junction (CEJ).^1^ GRs are rarely confined to a single tooth but often present as a generalized condition.^2^, ^3^ The treatment of multiple adjacent gingival recessions (MAGR) represents a demanding clinical scenario, due to the increased avascular recipient bed and extended surgical site, which increases the likelihood of encountering anatomical challenges such as a shallow vestibule, root prominence, uneven recession heights, and irregular keratinized tissue width.^4^ When MAGR affect the anterior maxilla—an area highly visible in the smile zone—clinical complexity is further amplified by high esthetic demands and the need to minimize the number of interventions to reduce patient discomfort.^2^, ^4^, ^5^

Among the several coronally advanced flap (CAF) designs proposed in the literature,^6^, ^7^, ^8^ the envelope CAF, introduced by Zucchelli and De Sanctis, has become one of the most widely adopted surgical techniques for the management of MAGR.^9^, ^10^ When used alone or in combination with a connective tissue graft (CTG), it offers highly predictable outcomes, with complete root coverage achieved in up to 80% of type 1 (RT1) GRs.^4^, ^5^ These results have demonstrated long‐term stability over 3–5 years.^11^, ^12^, ^13^, ^14^

In the original envelope CAF—known as the *lateral approach*—oblique papillary incisions converge toward an imaginary axis of rotation, typically at the tooth with the greatest recession, typically a canine or premolar.^9^ This design allows for the treatment of MAGR from the incisor to the first molar within a single quadrant.^9^ A subsequent variation, the *frontal approach*, was developed for bilateral MAGR involving the central and lateral incisors.^15^ In this modified approach, the flap's axis of rotation passes through the interincisal papilla. Notably, in contrast to the lateral approach, the midline papilla is not reflected but tunneled.^15^ However, when treating frontal and lateral MAGR in a single session, maintaining an intact midline papilla can limit coronal advancement of the flap, particularly in cases where a thick, full midline papilla restricts tissue mobilization beneath the contact point. Additionally, deep recessions on central incisors may induce mucosal folding along the gingival margins, challenging passive flap advancement. These factors may explain why the frontal approach has traditionally been limited to recession depths ≤ 2 mm.^15^, ^16^

To address this limitation, we propose a modification of the frontal envelope CAF, involving elevation of a V‐shaped split‐thickness surgical papilla at the midline. This adjustment facilitates flap release and enables coronal advancement beyond the CEJ, an essential criterion for complete root coverage. Our modification is designed to overcome the mechanical restrictions imposed by an intact midline papilla, thereby permitting passive, tension‐free flap advancement beyond the cementoenamel junction. Evidence indicates that favorable early healing and optimal clinical outcomes in gingival recession treatment depend on passive flap adaptation and tension‐free suturing.^17^ Two case reports were presented to illustrate the clinical application and outcomes of this technique.

## MATERIALS AND METHODS

The present case report follows the Case Report (CARE) guidelines.^18^

### Clinical presentation

**FIGURE 1:**
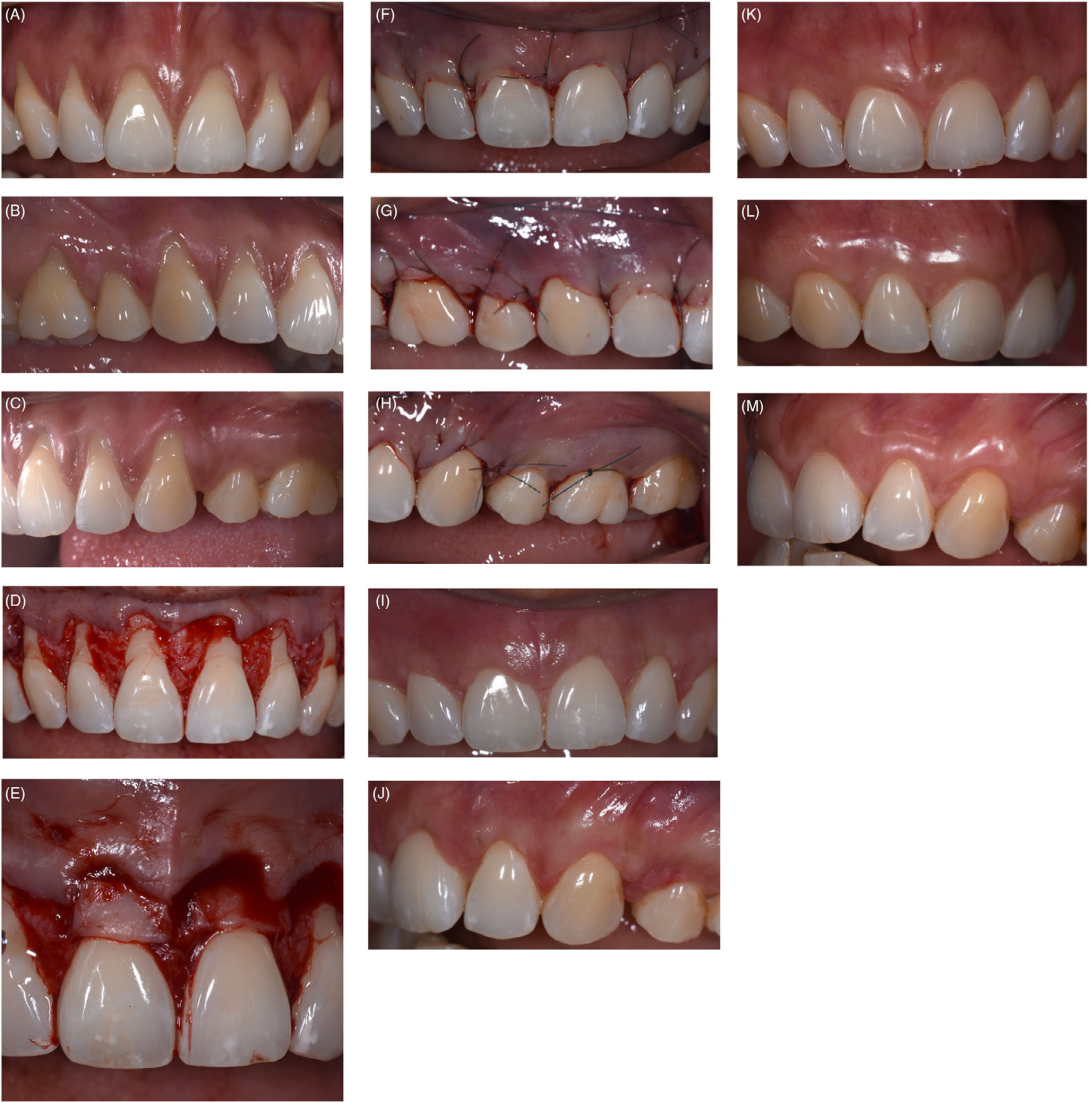
*Case 1*: (A) Frontal and (B, C) lateral views of the initial RT1/RT2 MAGR. Intraoperative views: flap design (D), flap elevation (E), site‐specific CTG placement (F), and flap closure (G). Postoperative views at 15 days (H–J) and 6 months (K–M), showing root coverage and tissue healing.

Case 1: A 34‐year‐old, systemically healthy, non‐smoking female patient presented to a University Hospital clinic in France, in July 2023. The chief complaint was esthetic concerns due to MAGR involving teeth 13 to 23 and 16. According to the 2017 World Workshop classification,^19^ the patient was diagnosed with RT1 GR, with recession depths ranging from 2 to 4 mm, a thick gingival phenotype with ≥2 mm of keratinized tissue (KT) and detectable CEJs. Slight root substance loss was observed, consistent with a history of traumatic toothbrushing (Figure 1). Following oral hygiene instructions and modification of brushing technique, the proposed surgical procedure was explained, and oral informed consent was obtained.

**FIGURE 2:**
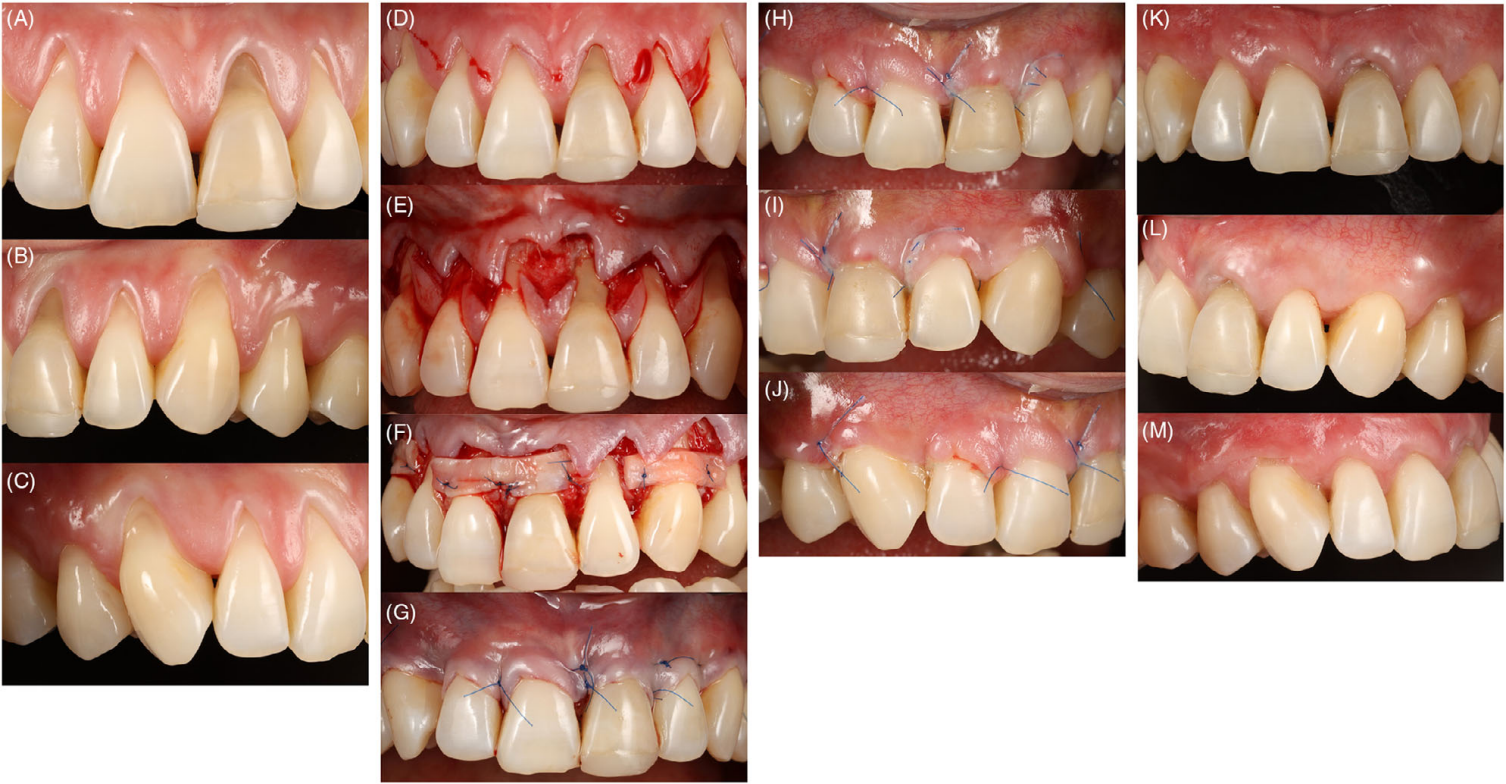
*Case 2*: (A) Frontal and (B, C) lateral views of initial RT1 MAGR. Intraoperative steps: flap elevation (D), site‐specific CTG placement (E), and flap closure (F–G). Follow‐up views at 15 days (H–J) and 18 months (K–M), showing stable complete root coverage and excellent tissue integration.

Case 2: A 46‐year‐old, systemically healthy, non‐smoking female patient presented to a private clinic in Spain, in October of 2024. The chief complaint was dentinal hypersensitivity and esthetic concerns due to the presence of MAGR involving teeth 14 to 24. According to the 2017 World Workshop classification,^19^ GRs were diagnosed as RT1 on teeth 23 and 14 and RT2 on teeth 13, 12, 11, 21, and 22. Recession depths ranged from 2 to 6 mm, and were combined with a thin gingival phenotype with ≤2 mm of keratinized tissue width (KTW), and detectable CEJs, along with root substance loss on teeth 13 and 23. Tooth 13 presented an incorrect dental position with slight vestibular rotation (Figure 2). Following oral hygiene instructions and modification of brushing technique, the proposed surgical procedure was explained, and oral informed consent was obtained.

### Surgical technique

**FIGURE 3:**
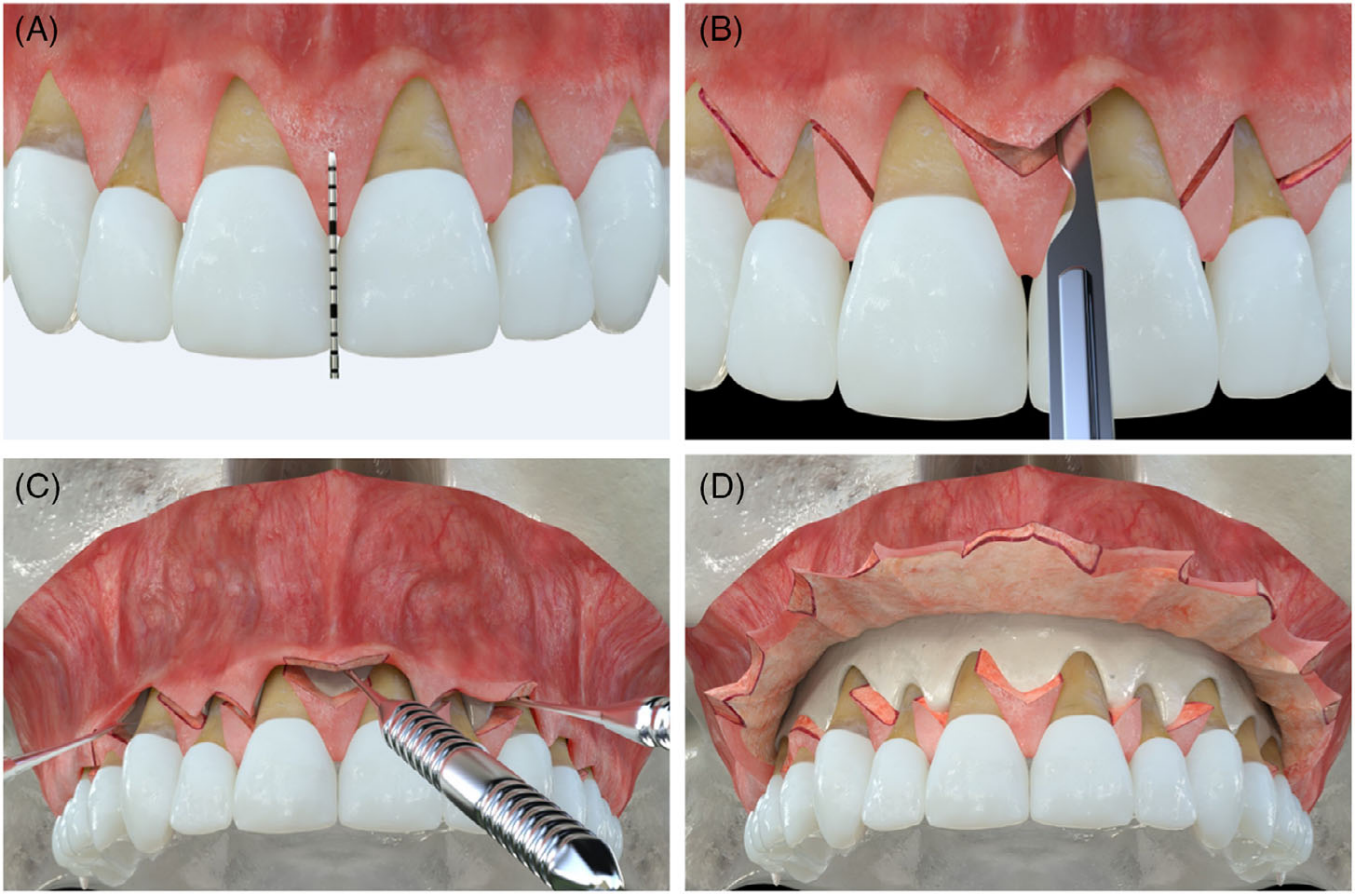
Schematic 3D illustrations showing step‐by‐step flap design and preparations.

The flap design was based on the frontal approach of the envelope CAF technique,^9^, ^15^ with slight modifications to enhance tension‐free coronal advancement of the midline papilla (Figure 3; Supporting Information S1). After administration of local anesthesia (2% articaine with 1:100,000 epinephrine), oblique papillary incisions converging toward the central midline were made in the interdental areas, according to the principles described by Zuchelli et al.^15^ In Case 2, a “false recession” was intentionally created on tooth 14 to balance the discrepancy with the recession on tooth 13.

A V‐shaped surgical papilla was created at the midline, by tracing two oblique papillary incisions from the mesial line angles of the gingival margins on the central incisors to the center of the interdental papilla the distance from the tip of the anatomical papilla. The height of the surgical papilla was determined by measuring from the tip of the anatomical papilla to a coronal point equal to the depth of the greater gingival recession on the central incisors plus 1 mm. The flap was elevated using a split–full–split technique in a coronal‐to‐apical direction: split‐thickness elevation at the surgical papillae, full‐thickness elevation apical to the recession margins extending past the mucogingival junction, and again double‐layer deep and superficial split‐thickness beyond the mucogingival line. Exposed root surface were mechanically instrumented using ultrasonic inserts (Insert H3, Acteon Satelec, Acteon Group, Mérignac, France) in conjunction with mini‐Gracey curettes. A free gingival graft harvested from the palate and de‐epithelialized extraorally for both cases. The obtained CTGs were selectively positioned and stabilized at the sites with the greatest gingival recession depths, lower gingival thickness, lack of keratinized tissue, and/or root concavities or malpositioning (teeth 11, 13, 16, and 23 in Case 1; teeth 13, 11, 21, and 23 in Case 2). Subsequently, the flap was coronally advanced to ensure passive, tension‐free coverage of the CTG and sutured using sling sutures. During flap advancement, the surgical papillae were positioned with slight distal rotation to conform to the underlying de‐epithelialized anatomical papillae. Only the midline surgical papilla was repositioned in a strictly vertical direction. For optimal flap positioning, initial fixation of the central papilla is recommended, followed by sequential suturing from the most distal to the most central sites. Coronal over‐displacement of the flap above the CEJ was intentionally performed to compensate for anticipated postoperative contraction. In Case 1, a horizontal double‐mattress suture was also placed at the vestibular base to reduce lip‐induced tension on the marginal portion of the flap (Figure 1). Postoperative instructions were provided to the patient, and antimicrobial mouthwash (0.12% chlorhexidine) along with analgesics (Ibuprofen 600 mg, three times daily for 5 days) were prescribed.

## RESULTS

Fifteen‐day postoperative follow‐up: In both cases, healing was uneventful and sutures were removed after 15 days. Postoperative morbidity was minimal, with no observed bleeding, infection, or flap dehiscence observed, and the gingival margins were well maintained in the desired positions (Figures 1 and 2). Eighteen‐month postoperative follow‐up (Case 1): At 18 months, complete (100%) and stable root coverage was observed across all treated sites. An improvement in soft tissue thickness was noted, along with excellent color match and tissue integration. The patient reported high satisfaction with the esthetic outcome. No scarring or loss of interdental papillae was observed at the incision sites (Figure 1, K–M).

Complete root coverage was achieved on teeth 22 and 23, with 90% to 95% coverage (residual recession of 0.5 to 1 mm) across the remaining teeth, accompanied by esthetic improvement and reduced dentinal hypersensitivity. The incomplete root coverage observed on teeth 11, 21, 12, and 13 may be attributed to the recession type (RT2) and/or tooth malpositioning (Figure 2, K–M).

## DISCUSSION

The frontal approach of the envelope CAF was originally indicated for shallow (≤ 2 mm) MAGR affecting central and lateral incisors with minimal (<1 mm) or no recession on the canines.^15^ In this approach, the midline papilla is typically tunneled, allowing the right and left segments of the flap to remain independent, making its unilateral application feasible when recessions are localized to one side only.^15^ However, when treating frontal and lateral MAGR in a single session, the limited coronal mobility of the tunneled midline papilla may act as a fixed point, particularly in clinical cases of RT1 recessions, compromising flap advancement in the anterior region or inducing mucosal folds in the gingival margin. In the present cases, elevating a V‐shaped surgical papilla instead of tunneling it, allowed for enhanced coronal displacement and eliminated this restriction in the anterior midline. Additionally, the V‐shaped incision facilitates the placement of the newly created surgical papillae into their future anatomical positions, as reported in previous flap designs.^7^, ^8^ The V‐shaped configuration mirrors the natural contour of the anatomical papilla, permitting precise adaptation and improved esthetic integration. Zucchelli et al. compared triangular and trapezoidal CAF designs, reporting similar root coverage outcomes but superior esthetic scores (patient VAS and professional ratings) for the triangular configuration, supporting our choice of incision design.^20^

The biological foundation of the envelope‐type CAF lies in maximizing vascular supply and flap stability. The anatomical interdental papilla plays a critical role in this context by serving both as a vascular bed and as a mechanical anchor for the most coronal part of the flap.^9^, ^21^ Although additional incisions might reduce local perfusion, angiographic studies have demonstrated adequate vascularization and healing of split‐thickness flaps repositioned over a well‐vascularized connective tissue bed.^17^, ^22^ This suggests that the V‐shaped surgical papilla receives sufficient revascularization from the underlying de‐epithelialized anatomical papilla.

From mechanical perspective, Wide and tall papillae enhance the potential to secure the flap coronally to the CEJ, a critical factor for achieving complete root coverage (CRC),^23^ while simultaneously ensuring an adequate vascular supply. Some reports have indicated a positive correlation between interdental papilla dimensions and the clinical success of CAF procedures.^24^, ^25^ One study reported that each 1 mm increase in papilla height and width was associated with a 17.5% and 8.4% improvement in mean root coverage, respectively.^24^ Based on this evidence, incision and elevation of the midline papilla may be preferred when anatomical papillae are wide and high, while narrow papillae may be better managed through tunneling. This approach aligns with recently proposed decision‐making criteria for selecting between CAF and tunnel in the treatment of MAGR,^26^ as well as with the “Tunneled‐CAF” (tCAF) technique, which selectively applies papilla incision or tunneling based on site‐specific anatomy of the interdental papilla.^27^

When the midline papilla is sufficiently wide, the use of two horizontal sling sutures, anchored respectively to the cingula of both central incisors, may enhance flap stability. The additional use of a horizontal internal mattress suture at the vestibular base of the papilla, intended to minimize lip‐induced tension, was adopted from previous reports.^9^ However, its effectiveness is primarily supported by clinical experience rather than comparative evidence.

Combining CAF with a CTG is associated with improved CRC rates and increased gingival thickness, which predicts long‐term marginal tissue stability.^28^, ^29^, ^30^, ^31^, ^32^ However, harvesting a CTG large enough to cover an entire arch may be technically demanding and could increase donor site morbidity. To address this, multiple small grafts were used to selectively augment sites requiring phenotype modification while minimizing patient morbidity. This approach allows treatment of several sites using smaller tissue sections, as each graft covers only the denuded root surface without extending into the interproximal area. Evidence indicates comparable clinical outcomes between smaller, selectively placed grafts and longer continuous grafts.^13^, ^14^ Moreover, smaller grafts are consistently associated with reduced postoperative discomfort, and a positive correlation between graft thickness and morbidity has been documented.^33^, ^34^ Therefore, minimizing graft dimensions can reduce patient discomfort while maintaining predictable clinical outcomes. Recent studies also demonstrate that site‐specific CTG placement in areas of thin gingival phenotype is equally effective, with no significant differences in CRC outcomes between grafted and non‐grafted sites over a 3‐year follow‐up period.^13^, ^14^ Moreover, in sites with cervical substance loss, CTG may also serve as a space maintainer, preventing the flap from collapsing into the defect.^35^ Current evidence indicates that in bilaminar techniques, graft thickness greater than 1 mm does not significantly enhance root coverage outcomes and may result in excess tissue volume and suboptimal esthetics.^33^, ^36^, ^37^ Anatomical studies report that the thickness of attached gingiva averages 1.25 ± 0.42 mm, typically greater in the maxilla than in the mandible.^38^, ^39^ Barootchi et al. demonstrated that a gingival thickness of approximately 1.46 mm, measured 6 months postoperatively, predicts stable long‐term outcomes over 10 years.^32^ Accordingly, the selective use of thin connective tissue grafts (CTGs) appears sufficient—particularly in the maxilla—to achieve effective site‐specific phenotype modification, while minimizing patient morbidity and optimizing esthetic outcomes. These principles guided CTG placement in the present cases, with priority given to teeth exhibiting root concavities, thin gingival phenotypes, or malpositioned teeth.

It should be acknowledged that the favorable outcomes observed in this case may not be attributed solely to the surgical technique. Site‐ and patient‐related factors, such as the presence of wide interdental papillae, a thick gingival phenotype, sufficient vestibular depth, non‐smoking status, and high compliance with pre‐ and postoperative instructions, likely contributed significantly to the treatment success.^2^, ^4^ Long‐term studies are needed to confirm the stability and generalizability of these results.

## CONCLUSIONS

Within the limitations of this case report, the modified coronally advanced flap combined with a V‐shaped surgical papilla appears to be an effective approach for managing bilateral maxillary MAGR extending beyond the incisors. Clinicians may consider this modification when enhanced coronal flap mobility is required to treat extensive frontal and lateral MAGR in a single session.

## AUTHOR CONTRIBUTIONS

**Kevimy Agossa**: Conceptualization (equal); data collection (equal); visualization (equal); original draft preparation (lead); writing—review and editing (equal). **Dino Calzavara**: Conceptualization (equal); data collection (equal); visualization (equal); writing—review and editing (equal). **Javier Calatrava**: Conceptualization (supporting); data collection (supporting); visualization (equal); writing—review and editing (equal). **Hom‐Lay Wang**: Validation (lead); writing—review and editing (equal); supervision (lead).

## CONFLICT OF INTEREST STATEMENT

The authors have no conflicts of interest to declare.

## PATIENT CONSENT

Before initiating the treatment, the expected outcomes were explained, and oral informed consent was obtained from both patients.

## REGISTRATION

The present clinical case report has been registered in the Open Science Framework (OSF) registry (N°: 5fbd8).

## Supporting information



Supporting Information
