# Supplementary material for: Modified coronally advanced flap for multiple anterior gingival recessions: Case report
Source: Clin Adv Periodontics. 2026 Jun 17;16(3):629–36. doi: 10.1002/cap.70049 (PMC13603046; doi:10.1002/cap.70049)
Supplement: Supplementary file 1 [file CAP-16-629-s001.docx]

**Supporting Information S1:** The link provides access to a supplemental video demonstrating the full surgical procedure. [https://youtu.be/oSVUDIYbWWg](https://urldefense.com/v3/__https://youtu.be/oSVUDIYbWWg__;!!N11eV2iwtfs!tyAdKz4vWsqqpQUniLlP0tD3iK2rGHBr2Uz0Ita8Kv4G184Ji__8zFJbRVxWIY17paGyATZ0_IE$)
